# A Case Series of E-cigarette or Vaping-Associated Lung Injury With a Review of Pathological and Radiological Findings

**DOI:** 10.7759/cureus.24822

**Published:** 2022-05-08

**Authors:** Seyedmohammad Pourshahid, Sara Khademolhosseini, Shahzad Hussain, Susanti R Ie, Maria Del Mar Cirino-Marcano, Sameh Aziz, Frank Biscardi, Mahtab Foroozesh

**Affiliations:** 1 Pulmonary and Critical Care, Virginia Tech Carilion School of Medicine, Roanoke, USA; 2 Internal Medicine, Icahn School of Medicine at Mount Sinai, Queens Hospital Center, New York, USA; 3 Pulmonary and Critical Care, Carilion Clinic, Roanoke, USA; 4 Internal Medicine and Critical Care, Carilion Clinic, Roanoke, USA

**Keywords:** e-cigarette and vaping product use-associated lung injury (evali), e-smoking, vaping, e-cigarettes, electronic cigarettes, outbreak, lung pathology, radiologic findings

## Abstract

There has been a recent outbreak of e-cigarette or vaping-associated lung injury (EVALI) but the exact pathophysiology remains unknown. Tetrahydrocannabinol (THC) and vitamin E derivates are the major components in vaping-generated aerosols that are associated with EVALI. So far, there is no standard treatment for EVALI. Most cases are treated with antibiotics and steroids. Counseling for smoking cessation is an integral part of care for EVALI patients. Referral to addiction medicine may be beneficial. Considering the nonspecific presenting symptoms and the growing popularity of vaping devices, providers need to consider EVALI in the differential diagnosis of bilateral patchy ground-glass opacities with respiratory, constitutional, or gastrointestinal symptoms in patients using e-cigarettes. Here, we present four EVALI cases and review the pertinent imaging and pathological findings.

## Introduction

E-cigarette and vaping products were initially introduced to the United States market in 2007 as a smoking cessation strategy, although many studies have since disproven this claim [1]. Nicotine, different flavorings, and additives such as tetrahydrocannabinol (THC) are typically present in the generated aerosols [2]. There are few regulations that control the quality and composition of ingredients used in e-cigarettes and vapes [3].

According to the primary case definition from the Centers for Disease Control and Prevention (CDC), e-cigarette or vaping-associated lung injury (EVALI) is defined as the presence of pulmonary infiltrates on a chest radiograph or ground-glass opacities (GGOs) on chest computed tomography (CT) with the use of e-cigarettes or vaping devices within the previous 90 days. Infectious causes, cardiac disease, connective tissue disorder, etc. must be ruled out as contributing to the pulmonary infiltrates or GGOs [4]. In 2019, the CDC announced an outbreak of EVALI, with a total of 2,558 EVALI cases requiring hospitalization and 60 deaths reported [5]. Chronic medical conditions such as heart disease, lung disease, or diabetes are risk factors associated with higher morbidity and mortality among EVALI patients [6]. The use of vitamin E additives and THC has been associated with EVALI outbreaks; however, data on causation remain insufficient [7]. The pathophysiology for EVALI and the harmful effects of vaping are not fully understood, and many of the specific characteristics are yet to be elucidated. Here, we present four cases of EVALI and review the radiological and pathological findings.

## Case presentation

Case 1

**Figure 1:**
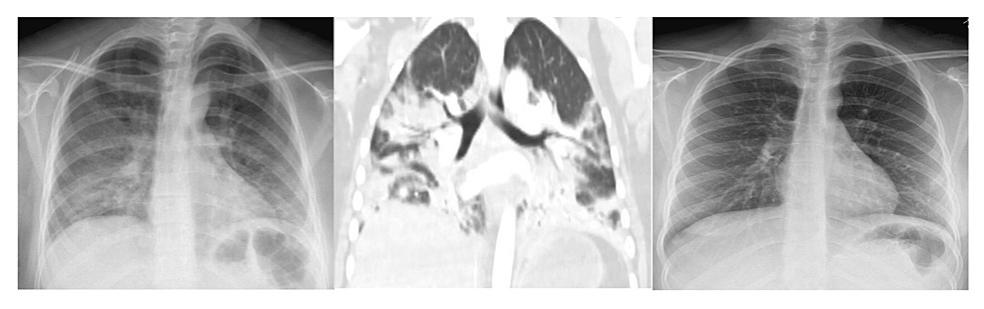
Left: CXR on presentation. Middle: Chest CT showing bilateral patchy consolidation and GGO on presentation. Right: Follow-up CXR after a month. CXR: chest X-ray; CT: computed tomography; GGOs: ground-glass opacity

**Figure 2:**
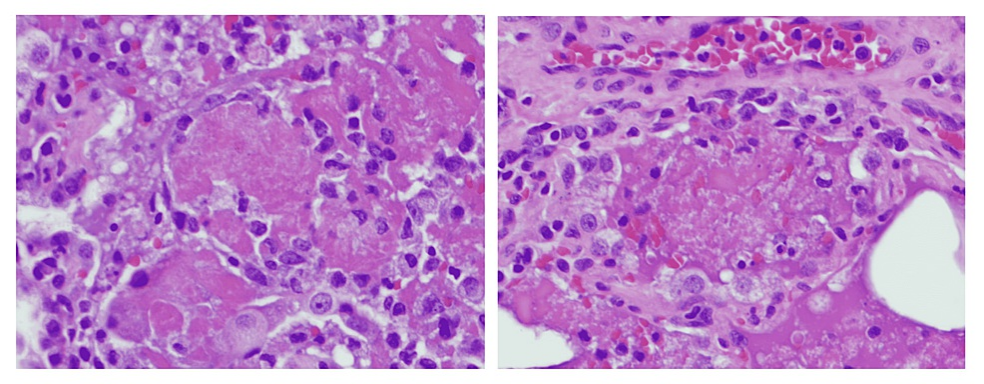
H&E staining of open lung biopsy in case 1. 400× magnification is demonstrating intra-alveolar fibrin, type 2 pneumocytes metaplasia, and intra-alveolar foamy macrophages on the left. On the right side, intra-alveolar fibrin, type 2 pneumocytes metaplasia, and pneumocyte vacuolization is depicted. H&E: hematoxylin and eosin

A 19-year-old female with a medical history of seizures and neurocysticercosis status post-stereotactic brain biopsy that was treated in her childhood presented with a complaint of worsening shortness of breath. She endorsed vaping for about two years and denied any drug use. She did not take any medication. Family history and environmental exposure were unremarkable. She had a recent emergency room visit for abdominal pain associated with nausea and vomiting a week prior to admission. On presentation, she was afebrile, with a heart rate of 134 beats per minute, respiratory rate of 16 breaths per minute, and blood pressure of 121/68 mmHg. Scattered end-expiratory wheezing and mild diffuse rhonchi were noted on auscultation. She denied any sick contacts. Chest X-ray demonstrated multifocal bilateral opacities (Figure 1), and labs were remarkable for a white blood cell (WBC) count of 13.8 K/µL. Rapid influenza was negative. She was empirically started on ceftriaxone and azithromycin for community-acquired pneumonia (CAP). Hypoxemia progressed and she required intubation, followed by mechanical ventilation. An echocardiogram revealed a normal left ventricular ejection fraction of 65-70%. Multiple blood cultures, sputum, and tracheal aspirate cultures, as well as a workup for atypical infections, including fungal and mycobacterial infections, were all negative. Human immunodeficiency virus (HIV) and immunoglobulin levels were unremarkable, and there was no evidence of an immunocompromised status. Vasculitis and connective tissue disease workup were negative as well. The patient underwent bronchoscopy with bronchoalveolar lavage (BAL) which showed only neutrophilic alveolitis (BAL had 1,075 red blood cells (RBCs) and 557 WBCs with a differential of 53% neutrophils, 35% monocytes, 10% lymphocytes, and 2% eosinophils). A surgical lung biopsy of the left lower lobe was performed. She finished a seven-day course of antibiotics (cefepime, levofloxacin, and linezolid) and was extubated two days after the open lung biopsy that revealed diffuse interstitial fibrosis associated with type 2 pneumocyte hyperplasia and patchy organizing pneumonia suggestive of acute lung injury and nonspecific interstitial pneumonia (NSIP) (Figure 2). NSIP can be one of the outcomes of acute lung injury.

She gradually improved and was discharged on room air 15 days after admission. She followed up in the pulmonary clinic a month after her discharge. By then, symptoms had resolved, and repeat chest imaging showed significant improvement in bilateral pulmonary opacities.

Case 2

**Figure 3:**
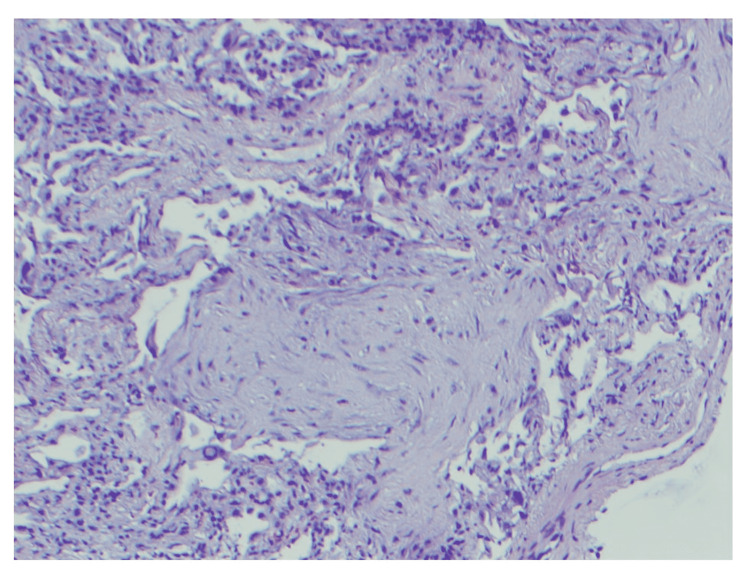
H&E staining of transbronchial biopsy from case 2: 100× magnification is demonstrating organizing fibrous tissue within the respiratory bronchiole which is suggestive of organizing pneumonia. H&E: hematoxylin and eosin

A 59-year-old man with a medical history significant for gout and anxiety presented with worsening shortness of breath. He was evaluated for similar symptoms five days prior to admission and took a course of doxycycline with no improvement. He had a smoking history of 36 pack-years but had transitioned to vaping two years before admission. On presentation, the patient was afebrile, with a heart rate of 110 beats per minute, respiratory rate of 20 breaths per minute, and oxygen saturation of 94% on room air with normal blood pressure. Diffusely scattered rhonchi were heard on auscultation bilaterally. Chest CT showed bilateral diffuse GGOs. He was empirically started on ceftriaxone and azithromycin for CAP. Blood culture, influenza, and workup for atypical pneumonia such as *Legionella *and *Mycoplasma* were all negative. Despite intravenous antibiotics for three days, the patient did not show any clinical improvement, and his respiratory status declined. The patient was transferred to the intensive care unit for worsening oxygen needs, requiring high-flow nasal cannula oxygen. The echocardiogram was unremarkable. He underwent bronchoscopy, and BAL had 610 WBCs (51% neutrophils, 27% lymphocytes, 12% eosinophils and 10% mononuclear cells). A transbronchial biopsy was consistent with organizing pneumonia with intra-alveolar foamy histiocytes adjacent to the alveolus with vacuolization of some type 2 pneumocytes (Figure 3). Findings were nonspecific, and considering the clinical context, EVALI was the top differential diagnosis. His case was discussed with a transplant center; however, due to unclear guidelines for transplant of possible EVALI cases and his recent smoking history, he was deemed not to be a transplant candidate. Fortunately, his breathing improved after a three-day steroid pulse (1 mg/kg/day). With improved oxygen requirements, he was discharged 17 days after admission on 8 L/minute of oxygen and prednisone at 60 mg oral daily and *Pneumocystis jirovecii* prophylaxis. A month after discharge, spirometry was normal and lung volumes showed a mixed obstructive and restrictive pattern with severely reduced diffusing capacity for carbon monoxide (DLCO). Arterial blood gas showed hypoxemia with an increased alveolar-arterial gradient. The patient continued abstinence from any smoking. He was weaned off oxygen and completed a prednisone taper four months after being discharged. The lung volumes and alveolar-arterial gradient continued to improve. Unfortunately, the patient had a smoking relapse six months after the hospital discharge. The follow-up pulmonary function test one year later revealed evidence of moderate obstruction with no bronchodilator response (Table 1).

**Table 1:** Pulmonary function trend of case 2 after discharge. FEV1: forced expiratory volume in the first second; FVC: forced vital capacity; TLC: total lung capacity; DLCO: diffusing capacity for carbon monoxide

Time after discharge	FEV1 (percent predicted)	FVC (percent predicted)	FEV1/FVC	TLC (percent predicted)	DLCO (percent predicted)
1 month	2.06 L (56)	2.61 L (55)	79	4.82 L (69)	8.62 mL/minute/mmHg (26)
4 months	2.75 L (75)	3.84 L (82)	72	5.94 L (84)	13.45 mL/minute/mmHg (41)
1 year	2.41 L (67)	3.83 L (82)	63	6.19 L (88)	16.51 mL/minute/mmHg (51)

Case 3

**Figure 4:**
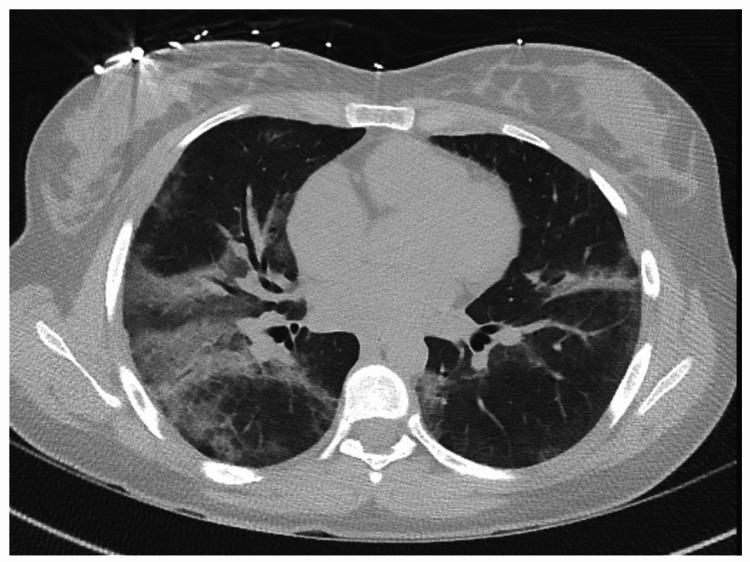
Chest CT of case 3 remarkable for bilateral patchy GGOs. CT: computed tomography; GGOs: ground-glass opacities

An 18-year-old woman with a medical history of obsessive-compulsive disorder and exercise-induced asthma presented with complaints of chest tightness and shortness of breath. The patient endorsed vaping and using marijuana daily for two years prior to admission. Vital signs on presentation included heart rate of 120 beats per minute, respiratory rate of 24 breaths per minute, and oxygen saturation of 93% on room air. Lung sounds were clear to auscultation. WBC count was 19.4 K/µL. CT chest showed patchy bilateral airspace GGOs (Figure 4) and no evidence of pulmonary embolism.

She was empirically started on ceftriaxone and azithromycin for CAP. Blood culture and sputum culture were negative, and workup for atypical infections was nondiagnostic. HIV test was negative. Considering the lack of clinical improvement and increasing oxygen requirements, antibiotics were broadened, and a repeat CT chest was done that showed waxing and waning of extensive bilateral GGOs.

**Figure 5:**
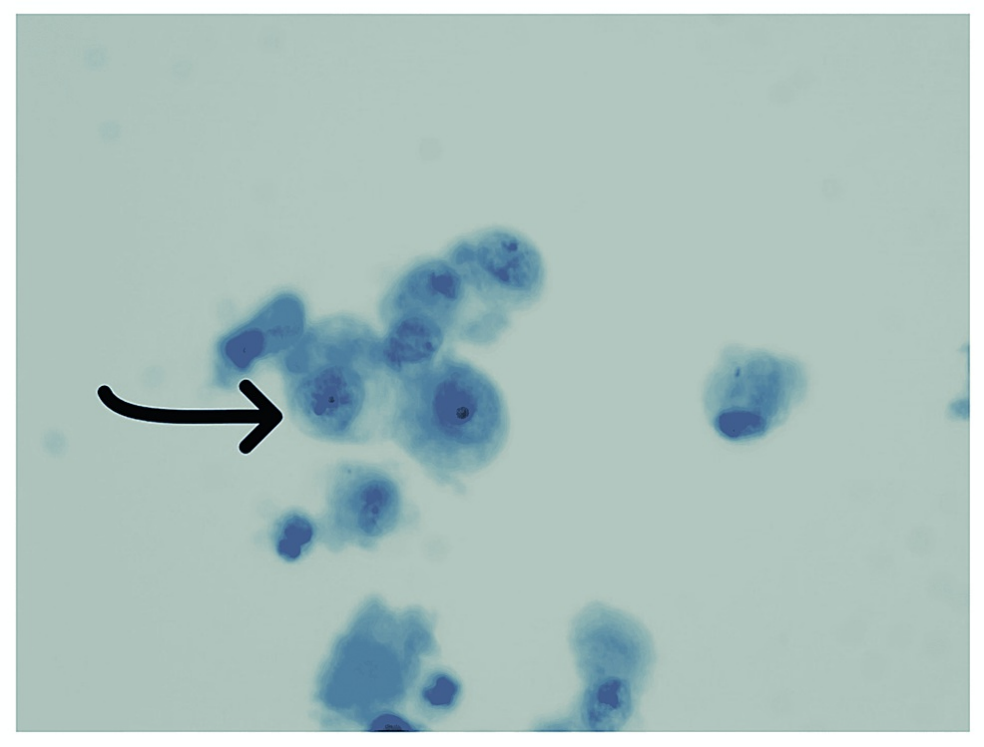
BAL of case 3, Papanicolaou stain. 600× magnification: demonstrating intra-alveolar foamy macrophages (arrow). BAL: bronchoalveolar lavage

**Figure 6:**
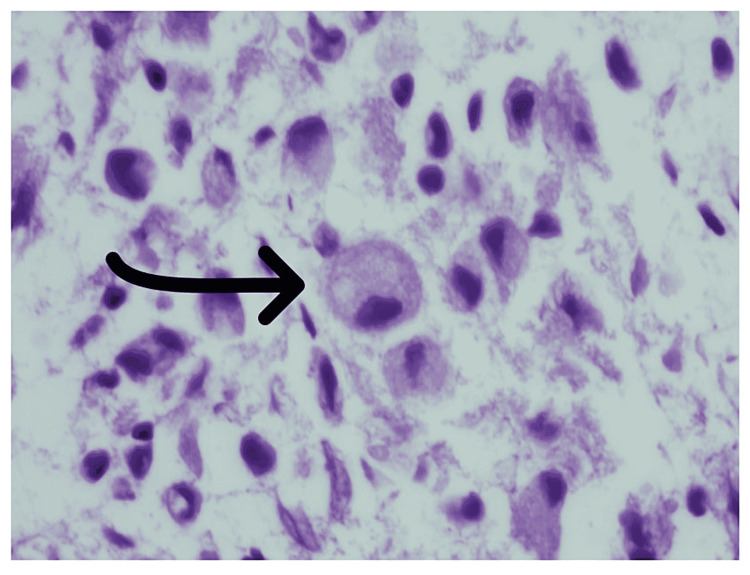
Cellblock from a transbronchial biopsy of case 3 (right) H&E. 600× magnification demonstrating intra-alveolar foamy macrophages (arrow). H&E: hematoxylin and eosin

She underwent bronchoscopy with BAL that showed neutrophilic and lymphocytic alveolitis (total WBCs of 782 with a differential of neutrophil 21%, lymphocyte 31%, monocyte 47%, and eosinophil 1%) with evidence of intra-alveolar macrophages (Figure 5). Cellblock from transbronchial biopsy revealed similar findings (Figure 6). Workup for fungal or mycobacterial pneumonia was negative. Antibiotics were stopped after seven days, and she was started on intravenous (IV) methylprednisolone with rapid resolution of symptoms. The patient was transitioned to oral prednisone, which was tapered over a course of 10 days.

Unfortunately, the patient did not follow up in the clinic and started to vape again a month after discharge. She presented to our hospital again with a worsening cough and shortness of breath. She had a fever of 103°F and was tachycardic to the 150s. She reported that she was admitted to another facility a week before and was treated with antibiotics for pneumonia. Her WBC count was 19.7 K/µL, and her chest X-ray was remarkable for diffuse GGOs bilaterally. She endorsed that her shortness of breath was resolved after the first hospital discharge until she started to vape again. Transthoracic echocardiogram revealed trace pericardial effusion and normal ejection fraction. Antibiotics were stopped and prednisone was given with rapid resolution of symptoms. She was discharged on a slower steroid taper with *Pneumocystis jirovecii* prophylaxis. She was counseled about the importance of abstinence from any smoking activities.

Case 4

A 34-year-old man with a history of tobacco abuse (smoking history of 16 pack-years and daily vaping for the past three years), polysubstance use (including cocaine, marijuana, and alcohol), depression, schizophrenia, and hepatitis C with cirrhosis was transferred from the inpatient psychiatry unit to the hospital for hypoxemia. The physical examination was otherwise unremarkable with clear breath sounds. Chest CT showed diffuse bilateral patchy GGOs and no pulmonary embolism. Considering the WBC count of 22.6 K/µL, the patient was started on cefepime, vancomycin, and azithromycin. Because of worsening oxygen requirements, the patient was intubated six days after being transferred to the hospital. A transthoracic echocardiogram revealed a ventricular ejection fraction of 55-60% with an estimated pulmonary artery systolic pressure of 23 mmHg. Left ventricle internal cavity size was mildly increased.

**Figure 7:**
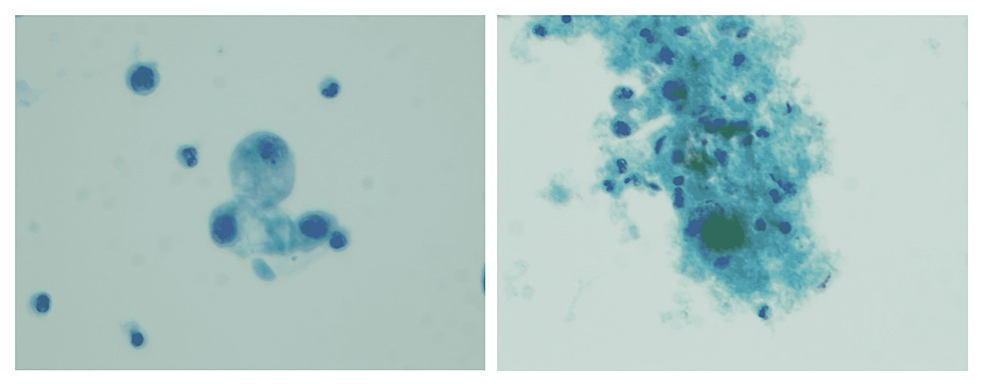
BAL from right upper lobe (left) Papanicolaou stain. 600× magnification: intra-alveolar foamy macrophages. BAL from right middle lobe (right) Papanicolaou stain. 400× magnification: intra-alveolar foamy macrophages with intracytoplasmic pigments. BAL: bronchoalveolar lavage

**Figure 8:**
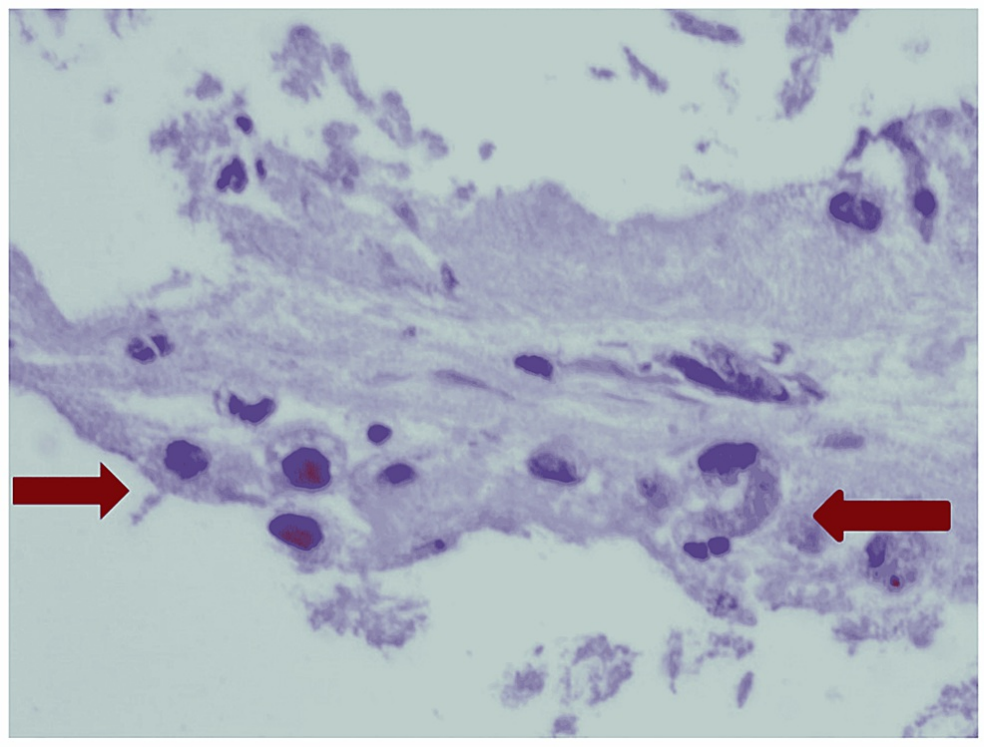
Cellblock from case 4. H&E. 600× magnification: interalveolar macrophages with intracytoplasmic pigmentation on the right and vacuolated cytoplasm on the left. H&E: hematoxylin and eosin

Cultures were negative, and an IV steroid was added. Bronchoscopy with BAL showed neutrophil-predominant lavage (WBC 331, neutrophil 57%, lymphocyte 13%, monocyte 30%) without any growth on cultures. BAL cytological assay showed intra-alveolar foamy macrophages (Figure 7). A transbronchial biopsy was remarkable for intra-alveolar macrophages with intracytoplasmic pigmentation and vacuolated cytoplasm (Figure 8). The course was complicated with a spontaneous pneumothorax requiring chest tube placement. After six days of mechanical ventilation, he was successfully extubated with the removal of the chest tube. Respiratory status continued to improve, and the patient was discharged on oxygen at 2 L/minute. The patient was discharged on a slow prednisone taper over a period of two months with pulmonary clinic follow-up. He was counseled about the importance of smoking and vaping cessation.

## Discussion

The limited understanding of EVALI is primarily derived from a few patient case series and reports [8]. The majority of EVALI patients are young adults and generally healthy before the presentation. The duration of vaping widely varies from weeks to many years [9]. The majority of patients reported at least one or more symptoms, including respiratory (97%), constitutional (90%), or gastrointestinal (90%) symptoms. Cough, dyspnea, fever, nausea, and vomiting are the most common presenting symptoms [8]. Leukocytosis was the most common laboratory abnormality [8,9].

Multifocal, diffuse GGOs were reported in almost all the cases, but consolidation, interseptal thickening, and tree in bud patterns have been reported as well [10,11]. Aberegg et al. [8] reported airway wall thickening in 81% and subpleural sparing in 39% of the cases, but no specific pattern of radiographic location has been agreed upon in the review of cases [9].

Nonspecific acute lung injury with various degrees of diffuse alveolar damage and organizing pneumonia were the most common findings in lung biopsy. Foamy macrophages, interstitial edema, fibrinous exudate, and hyaline membrane were common histopathologic findings [12,13]. Lipid-laden macrophages (LLM) were present in more than 80% of reported BAL studies; however, the clinical significance is still unclear, as this could be seen in various diseases such as lipoid pneumonia and amiodarone toxicity. The presence of LLM is proposed as a possible marker of EVALI or a marker of the causative agent for EVALI, but further studies are required [8,12,13]. In our patients, lymphocytic and neutrophilic alveolitis was noted. EVALI is considered a diagnosis of exclusion, and bronchoscopy is extremely valuable in ruling out the alternative causes [14].

Treatment is not yet standardized but the overwhelming majority of the cases receive a course of antibiotics and steroids. There is no consensus on the dosing and duration of steroid therapies [8]. Although most patients improve with the above-mentioned treatments, some cases may continue to progress. There are reports of extracorporeal membrane oxygenation utilization as a bridge to recovery and very few cases of lung transplant for EVALI with end-stage lung disease [15]. Currently, there are few transplant centers that accept such patients. This is a topic that requires more attention considering the majority of EVALI patients are young and relatively healthy.

There are a few reports of readmission or possible relapse of EVALI, with the majority of cases occurring in patients who resumed vaping after discharge, as seen in our third case [8]. Advising patients to stop using e-cigarettes and vaping is an integral part of care for EVALI patients. Therefore, behavioral or addiction counseling should be offered as well as medications that may help with the cessation of vaping and using e-cigarettes. Concomitant cannabis use may significantly increase the chance of relapse [16].

In this case series, we presented four patients who were hospitalized with respiratory and constitutional symptoms. Patient number three had preceding gastrointestinal symptoms for a week prior to admission for respiratory issues. All four patients had leukocytosis and patchy bilateral GGOs on chest CT. All received empiric antibiotics initially and all except for patient number one received steroids. The dose and duration of steroid therapy were variable. Three out of four had neutrophil predominant BAL. Organizing pneumonia, acute lung injury, and foamy macrophages were the predominant patterns observed on the biopsy. Patient number two was evaluated and rejected by a transplant center, but fortunately recovered with high-dose steroids. He had a follow-up PFT one year later with evidence of improvement in TLC and DLCO; however, he later developed an obstructive pattern on his PFTs that could be related to a relapse of smoking. Patient number three had a recurrence of EVALI after two months likely due to a relapse of vaping.

## Conclusions

We present four cases of EVALI and reviewed the related imaging and pathological findings. As the number of EVALI cases has risen significantly, it is crucial to raise awareness. There are many unknown aspects of this disease, including pathophysiology and treatment options, that requires further studies. Considering the nonspecific presenting symptoms and growing popularity of vaping devices, providers need to consider EVALI in the differential diagnosis of bilateral patchy GGOs and respiratory, constitutional, or gastrointestinal symptoms in patients who use e-cigarettes.
